# Global Accreditation Strategies in Health Management Education

**DOI:** 10.3389/fpubh.2019.00012

**Published:** 2019-02-18

**Authors:** Daniel J. West, Bernardo Ramirez, Gary Filerman, Anthony Stanowski, Otar Vasadze, Ana Marie Malik, Francesco Yepes, Vladimir Krcmery

**Affiliations:** ^1^Department of Health Administration & Human Resources, The University of Scranton, Scranton, PA, United States; ^2^Department of Health Management and Informatics, University of Central Florida, Orlando, FL, United States; ^3^Atlas Research Foundation, McLean, VA, United States; ^4^Commission on Accreditation of Healthcare Management Education, Rockville, MD, United States; ^5^Department of Health Administration, University of Georgia, Tbilisi, Georgia; ^6^Coordinator FGV-Saude, Getulio Vargas Foundation, São Paulo, Brazil; ^7^Postgraduate Programs in Health Administration & Public Health, Pontific Xavierian University, Bogota, Colombia; ^8^School of Tropical Medicine & Social Work, St. Elizabeth University, Bratislava, Slovakia

**Keywords:** accreditation, CAHME, competency, networks, partnerships, strategy

## Historical Background

The Association of University Programs in Health Administration (AUPHA) was founded with the support of the W.K. Kellogg Foundation (WKKF) in 1948 as a strategy to develop specific competencies and professional identity of hospital administrators to improve the performance of the rapidly growing and increasing complexity of hospitals throughout the United States and Canada (1).

Accreditation of graduate programs started in 1968 with the establishment of the Accrediting Commission on Graduate Education for Hospital Administration (ACGEHA), and in 1975 was changed to the Accrediting Commission on Education for Health Services Administration (ACEHSA). In 2004, the organization was renamed to the current Commission on Accreditation of Healthcare Management Education (CAHME) (2).

In the 1950s and 1960s the WKKF began the support of health (hospital) administration education in Latin America. In 1964 the AUPHA received a grant from the WKKF to further expand the international activities of the Association. By 1966 AUPHA conducted the first Latin American Conference in Hospital Administration Education in Bogota, Colombia with the participation of faculty and program directors from 10 countries (3). By the mid 1970's and early 1980's a network of centers of excellence had been developed in several countries of Latin America with more than 10 years committed support from the Kellogg Foundation (4). By the mid 1980's AUPHA and its members had developed a very significant network that extended with partnerships across the globe.

Between 1985 and the year 1997, AUPHA entered into a cooperative agreement with the United States Agency for International Development (USAID) aimed at strengthening health administration education in Latin America. From 1994 to the year 2000 AUPHA participated in a separate Cooperative agreements and grants with USAID and other donors to create and develop health management education partnerships and executive workshops in most countries of the Newly Independent States in the Former Soviet Union (5) and Eastern Europe (6–8). During this period, faculty interested in international healthcare and education started and developed a Global Healthcare Management Faculty Forum within the programmatic activities of AUPHA. This forum has been very active in mapping out and supporting global healthcare management education curriculum, competencies, training materials. It supports many workshops and partnerships that have contributed to the advancement of excellence and a global perspective in the member programs of AUPHA and abroad (9, 10).

Because of these activities and intensive partnership interaction the academic community and the field have been preparing the climate for more structured accreditation and/or certification initiatives (11).

## Global Accreditation

The concept or idea of international/global accreditation has been a topic of discussion among accrediting agencies and organizations. The Council for Higher Education Accreditation (CHEA) has produced several policy papers extending quality review of higher education into a larger global context. A borderless world in higher education provides mobility of student and faculty to enhance scientific research and to redesign undergraduate, postgraduate, and doctoral education. Within this larger context of globalization, there is an opportunity for existing accrediting organizations to provide accreditation and certification activities that impact the current and future development of professional health care leaders and quality of care. The demand for effective and efficient leadership is a global matter that has drawn the attention of the International Hospital Federation (IHF) in the development of a competency directory (12). Countries in all regions of the world are concerned with leadership, governance, quality of care, the patient experience, and access to care.

In the European Union in 1999, the Bologna Declaration was passed with a purpose to adapt European Higher Education and Research to social changes and scientific advancements. The same is true of organizational changes in the Asia-Pacific region and Africa. Joint Commission International has developed a strong presence in many countries focusing on quality and improved health care services and systems. Increasing international collaboration has been noted in practices associated with the Association to Advance Collegiate Schools of Business (AACSB) and the European Foundation for Management Development (EFMD). Regional quality assurance associations and agencies have been developed such as the European Association for Quality Assurance in Higher Education (EAQA); Asia-Pacific Quality Network (APQN); Arab Network for Quality Assurance in Higher Education and the African Quality Assurance Network. There has been a corresponding growth since 2000 in the number of national accrediting agencies in higher education.

International or global accreditation has been a topic of discussion for both CAHME and AUPHA. The AUPHA Global Healthcare Management Faculty Forum has been instrumental in developing a body of knowledge, identifying competencies, and advancing global perspective on health management education (HME). Recent thinking around global accreditation occurred in March 2016 during the American College of Healthcare Executives (ACHE) Annual Congress when CAHME assembled leaders in healthcare and education to discuss the future of graduate HME. The White Paper created as a by-product of this meeting served as a resource in the development of a 3-year plan. One of the core initiatives included global accreditation. Efforts have been noted with other accrediting organizations in the United States that have taken on opportunities for international accreditation given the global perspective and relevance of most health-related professions (13). The Association to Advance Collegiate Schools of Business (AACSB) is now accrediting business administration programs and the Council on Education of Public Health (CEPH) has been offering global accreditation since 2006 (14). So, there is a precedence set for US accrediting agencies to offer accreditation opportunities globally. In addition to US accreditation, the Council for Business Schools and Programs (ACBSP) and the European Management Development Network (EMDN) out of Brussels, England is also providing accreditation in 80+ countries.

## Applied Research

The mission of CAHME is to advance the quality of graduate healthcare management education. CAHME currently accredits 102 programs in the United States and Canada. With funding through the ARAMARK Charitable Fund, two research studies (Phase I and Phase II) were authorized to gather specific information and answer important questions impacting graduate health management education in the United States. The studies also had a domestic and international component in the methodology and purpose. These two studies had some limitations such as the fact that the target respondents were program directors and number of respondents to the follow up telephone survey (65%) on the phase II. Also keeping up with the current and rapidly growing activities and dynamic progress and evolution of global healthcare management education activities. Also, the relative difficulty to keep up with the development of the field around the world, even with the rapid growth of communication technology.

Phases III and IV have not received financial support from a specific donor organization and have been the result of a collaborative interaction of several interested faculty and CAHME and AUPHA member programs that are very active in the global healthcare management education arena. Other health management practitioner organizations, such as IHF, ACHE, and several national similar organizations have joined this partnership effort in support of the professionalization of the healthcare management profession.

### Phase I Study

The Phase I study was conducted in 2011 and was structured to examine the supply and demand for professional trained health care administrators in 16 countries; provide a summary of health systems; assess the extent of international healthcare management education activities of CAHME accredited programs and describe involvement in international health administration education (15). The 16 countries included Austria, Brazil, Chile, France, India, Israel, Mexico, Philippines, Saudi Arabia, Singapore, South Africa, Spain, Sweden, Turkey, and the United Kingdom. The Phase II study conducted in 2012 added 6 additional countries including Germany, Ireland, Czech Republic, South Korea, Netherlands, and Colombia bringing to a total 22 countries that were examined in terms of educational and accreditation activities (16). The number of universities identified in the study were 208 that offered health management related programs at different academic levels (from doctoral to undergraduate, including specializations and certificate programs). There were 142 master's degree programs offered and 20 universities that had multiple master's degrees. There are many diverse names and school affiliations and similar to the US and Canada, there is no unified degree conferred for health administration.

The Phase I study revealed several key findings including the following:

- Approximately 30% of CAHME accredited programs have international involvement of some type;
- University-based partnership models have been identified as a venue for different types of educational endeavors from courses, workshops/seminars, short courses, certification courses, and lectures;
- Approximately 30% of program directors reported that their graduate programs provide study abroad, student exchanges, faculty exchanges, online graduate courses, and service learning opportunities abroad;
- CAHME programs are active in many countries but the focus seems to be on Asia, Middle Eastern, and Western European countries.

### Phase II Study

The Phase II survey focused on specific international management areas: global centers, research, courses, study abroad, and partnerships (16). The Phase II study also developed a strategy to implement international demonstration site visits using the 2013 CAHME accreditation criteria (17). The following are among key findings of the Phase II survey:

- 42% of surveyed programs offer study abroad;
- 69% of programs have faculty involved in some type international research;
- 38% of the graduate programs offer global health management courses;
- 46% of programs reported having international partnerships with universities in another country.

The two studies are nicely summarized in an article published by West et al. (16) in the International Hospital Federation journal of World Hospitals and Health Services. The article entitled “Leadership in Globalization: Research in Health Management Education” offers suggestions relative to graduate education and the impact of globalization. As noted by the authors

“…. Globalization of health management education continues to mature in response to the need to ensure access to health care services in every country, addressing the demand for cost effective care and improving quality of care. The issues of costs, access, and quality are global and are driving the need for trained leaders and managers who can improve the performance of health systems and in particular effectively manage care across public and private sectors.” (p. 16)

### Phase III Study

CAHME accreditation standards and criteria have been used with healthcare management programs at universities outside of the USA and Canada on an informed basis to provide a structure for process improvement. CAHME 2008 and 2013 standards and criteria were used and applied as a first demonstration site at St. Elizabeth University, Bratislava, Slovak Republic using an international site visit team from Italy, Austria, Hungry, and the USA. The majority of the 35 criteria were determined to be applicable and useful in university-wide accreditation. With this successful first experience St. Elizabeth University has been using successfully some of the CAHME criteria and process with educational projects that they conduct in several countries in Africa. A modified version of the 2008 CAHME criteria were used at the University of Georgia, Tbilisi, Republic of Georgia to help with the development of a graduate MHA curriculum. The authors have also applied CAHME 2013 criteria as a basis for educational efforts in Mexico to train trainers and healthcare managers for the accreditation efforts of health care organizations in that country. Finally, a survey team from Brazil and the USA used CAHME criterion 2013 at Javeriana University in Bogota, Columbia with success. The Phase III applications provided additional evidence for using international site visit teams and competency-based criterion with a quality improvement process. These demonstration projects supported the further development of global accreditation in HME by CAHME.

### Phase IV Study

Based on the success of the Phase III of the study, the authors worked with the CAHME leadership team to further develop a strategy for global accreditation that would incorporate the previous years' experience with the concurrent ideas in the field. The results of these efforts have facilitated the current efforts by CAHME at global accreditation that involves using a Candidacy Process and Mentorship Program to enable universities outside of the USA and Canada to apply for CAHME accreditation. The CAHME Board of Directors in November 2017 approved a Global Advisory Council (GAC) to work directly with the CAHME President and CEO to implement global accreditation in graduate health management education. As of July 2018, there are five universities expressing an interest in CAHME accreditation and with two of these programs completing an eligibility statement.

## CAHME Framework

The CAHME mission is to “serve the public interest by advancing the quality of health care management education.” The values that CAHME holds firmly includes integrity, excellence, transparency, fairness, and recognition. The CAHME corporate members in partnership with the academic community (accredited graduate programs) ensure that graduate healthcare management curricula reflect the needed industry competencies.

In order to advance the mission of CAHME on a global scale, the vision is to utilize the existing structure and accreditation criteria of CAHME to offer accreditation to universities with specific graduate programs in health management outside of the United States and Canada. The vision further encourages cooperation between CAHME and AUPHA in the area of globalization. Accredited programs are composed of faculty who have an interest and established relationships with universities outside of the United States. The vision is to utilize faculty who have established international partnerships as a way of beginning to engage global accreditation. Strong working relationships with existing national accrediting organizations and professional associations is appropriate. CAHME has leveraged global relationships with IHF. CAHME will work to develop collaborative relationships with SHAPE (18) (Society for Health Administration Programs in Education) (19, 20); ASPHER (Association of School of Public Health in European Region); and CLADEA (Latin American Council of Management Schools). The vision includes offering global accreditation that has cultural relevancy; advancing the CAHME brand and standards; advancing the CAHME mission; utilizing competencies that drive standards; and working with universities that have established relationships with other global regions. The CAHME Mentorship Circle enables and encourages accredited programs to help universities outside the USA and Canada to pursue CAHME accreditation.

## Organizational Structures

**Figure 1:**
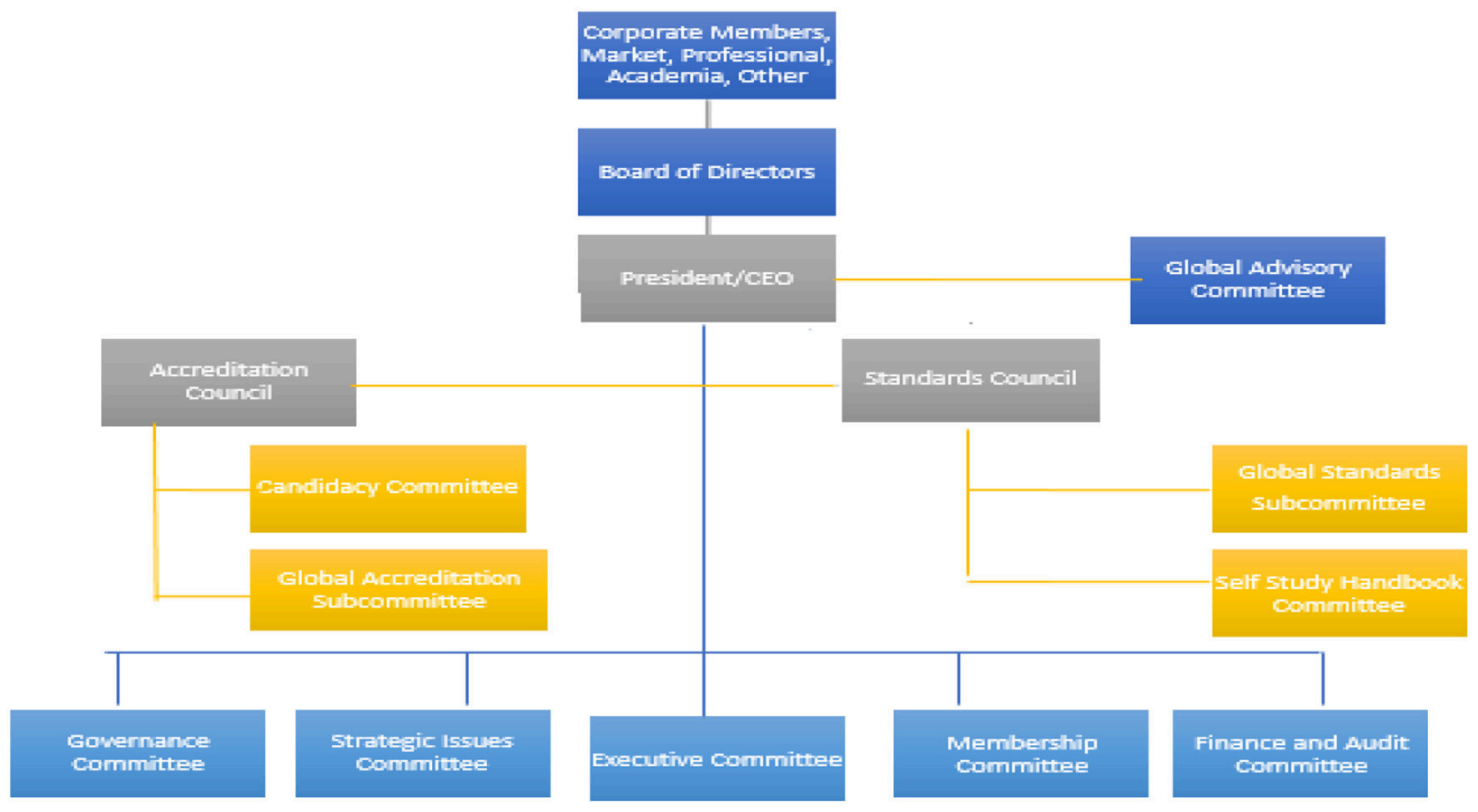
Proposed structure to accommodate Global Accreditation. Source: Global Accreditation in Health Management Education: Concept Paper (21).

Global accreditation will utilize the most recent (2017) CAHME Accreditation Standards and Criteria. A Global Advisory Council (GAC) has been established to work directly with the President and CEO. Global accreditation will necessitate that CAHME access and utilize faculty from CAHME accredited programs who have accreditation experience and partnerships with universities outside of the United States and Canada. CAHME needs faculty and professionals with global experiences to work with the CEO, Accreditation Council & Standards Council (Figure 1).

CAHME accreditation will have merit to select and engage universities who embrace program specific accreditation, an ongoing process of quality improvement guided by academic peers and practitioners, and who desire CAHME recognition (19). This is similar to AACSB, CEPH, and Joint Commission International (JCI). The Global Advisory Council can recommend appropriate modifications to accommodate global universities to the Accreditation Council and Standards Council. The existing Candidacy Committee will be used to work with programs seeking CAHME accreditation. The Global Advisory Council will seek members with international training and experiences to serve on site visit teams. As more programs are accredited globally, these programs will be recruited to serve on future accreditation site visits. In this way, global accreditation will grow slowly and expand as the demand increases.

CAHME will utilize a university-based partnership model where current CAHME accredited programs serve as an international university partner in the CAHME process. The CAHME model of a peer reviewed, voluntary, public process will be used. Academic freedom and shared governance remain essential eligibility criteria. The “CAHME Mentorship Circle” as previously described provides an excellent platform for building global partnerships and models of collaboration.

## Strategic Design Considerations

Moving forward with global accreditation will require certain innovations and adaptations by CAHME to ensure successful implementation. The following list of considerations will be used to implement the organization structure and strategic initiatives:

1. Use 2017 CAHME Eligibility Criteria and Accreditation Standards accounting for cultural and national variations.
2. Utilize existing university-based partnerships from CAHME accredited programs as a way of encouraging universities to pursue accreditation and advance candidacy.
3. Appoint established CAHME accredited program faculty and professionals with global knowledge and experience to the Global Advisory Council. This GAC will work with the Accreditation Council and the Standards Council to adjust/update standards.
4. Create a Global Health Fellows Program similar to the existing CAHME fellows but with a different focus: to assist the President & CEO with research and background, developing policy and procedures; and coordinating site visits.
5. Develop cooperation with national organizations to help support the concept of global accreditation (EHMA, SHAPE, ASPHER, & EURAM).
6. Utilize experiences of other accreditation agencies (e.g., CEPH, AACSB, ACBSP, etc.).
7. Utilize the resources and support of the IHF including but not limited to the IHF Competency Directory.
8. Use international faculty on site visit teams who have a global accreditation background and have specific knowledge of the culture of countries.
9. Align relevant activities with the AUPHA Global Leadership Committee and the Global Healthcare Management Faculty Forum.
10. Operationalize the Global Accreditation Subcommittee and the Global Standards Subcommittee of the GAC.
11. Use the existing CAHME fee structure, which includes the pass through of direct travel costs.
12. Participate with the Healthcare Management Strategic Interest Group (HM-SIG) of the IHF, the EURAM Healthcare Management Track of the Public and Nonprofit Management Special Interest Group (SIG), and other similar current or future identified groups. Also utilize the experience of US and Canadian universities and Faculty that actively participate in these groups.
13. Advance the CAHME Academic and Practitioner Model.
14. Utilize the CAHME Membership Circle strategy to help universities in the Candidacy Committee Process.

## Conclusion

The creation of global networks among universities is not new. The Magna Charta Universitatum is a document that was signed by 388 rectors (university presidents) from all over Europe and beyond on September 18, 1988, also known as the Bologna Accord. It contains principles of academic freedom and institutional autonomy as a guideline for good governance and self-understanding. Today there are 825 universities from 85 countries. The concept endorses the importance of collaboration in study, teaching and research. Other strategic initiatives include Faculty Fulbright Scholars, study abroad programs, language immersion programs, faculty directed research and public-private university-based partnerships.

There is increased interest among CAHME accredited program faculty for involvement with the international community. Other organizations such as the European Academy of Management (EURAM) could be important in this process. CAHME faculty are supported by the International Hospital Federation (IHF), especially the health management (HM) strategic interest group (SIG). The IHF has received significant support from ACHE and other international bodies in developing and advertising the IHF Competency Directory. The Global Consortium for Healthcare Management Professionalization has worked since January 2013 with “the shared aim of professionalizing the leadership and management of health systems to improve patient care globally” (12, p. 3). Several CAHME accredited performance come from universities that have a major effect on globalization of research and graduate education. There is reason to believe that many countries may support the idea of global accreditation.

## Author's Note

This manuscript has not been submitted for publication with another journal. The ideas expressed in this manuscript have been implemented in various phases, and are original ideas developed and used by the authors related to CAHME and global accreditation.

## Author Contributions

DW and VK: original research; BR: research in Mexico; GF: Aramark studies; AS: CAHME concept; OV, AM, and FY: site visit survey.

### Conflict of Interest Statement

The authors declare financial support from the Aramark Foundation via a contract with CAHME to conduct PHASE I and PHASE II survey research. There are no perceived conflicts of interest. All the concepts and ideas expressed and discussed are original and conceived by the authors.
